# The Association Between Phase Angle Decline and Functional Recovery Following Periacetabular Osteotomy: A One-Year Prospective Evaluation

**DOI:** 10.3390/jcm15062161

**Published:** 2026-03-12

**Authors:** Daisuke Homma, Norio Imai, Dai Miyasaka, Moeko Yamato, Tsubasa Sugahara, Masafumi Ishisaki, Mie Yamada, Hayato Suzuki, Yoji Horigome, Atsushi Sakagami, Yoichiro Dohmae, Naoto Endo, Izumi Minato, Hiroyuki Kawashima

**Affiliations:** 1 Division of Orthopaedic Surgery, Graduate School of Medical and Dental Sciences, Niigata University, 757 Asahimachi-dori Ichiban-cho, Chuo-ku, Niigata 951-8510, Japan; inskawa@med.niigata-u.ac.jp; 2 Department of Rehabilitation, Niigata Bandai Hospital, 2-2-8 Yachiyo, Chuo-ku, Niigata 950-0909, Japan; yayamamatoto.pt@gmail.com (M.Y.); sugahara.t24@gmail.com (T.S.); isisaki_rinkorh@yahoo.co.jp (M.I.); hykwme7532@gmail.com (M.Y.); 3 Division of Comprehensive Musculoskeletal Medicine, Graduate School of Medical and Dental Sciences, Niigata University, 757 Asahimachi-dori Ichiban-cho, Chuo-ku, Niigata 951-8510, Japan; imainorio2001@yahoo.co.jp (N.I.); yhori@med.niigata-u.ac.jp (Y.H.); 4 Division of Orthopaedic Surgery, Niigata Bandai Hospital, 2-2-8 Yachiyo, Chuo-ku, Niigata 950-0909, Japan; miyasakad@yahoo.co.jp (D.M.); always.smiling0418@gmail.com (A.S.); y-dohmae@ngt-rinkohc.jp (Y.D.); 5 Division of Orthopaedic Surgery, Tachikawa General Hospital, 24-1 Asahioka, Nagaoka 940-8621, Japan; hayato_suzuki614@yahoo.co.jp; 6 Department of Orthopedic Surgery, Saiseikai Niigata Kenoh Hospital, 5001-1 Kamisugoro, Sanjo 955-0091, Japan; nendo120@gmail.com; 7 Division of Orthopaedic Surgery, Niigata Rinko Hospital, 1-114-3 Momoyamacho, Higashi-ku, Niigata 950-0051, Japan; imrinkoh@yahoo.co.jp

**Keywords:** osteoarthritis of the hip, curved periacetabular osteotomy, periacetabular osteotomy, phase angle

## Abstract

**Background/Objectives**: Curved periacetabular osteotomy (CPO) is an effective joint-preserving procedure for osteoarthritis of the hip; however, postoperative weight-bearing restrictions may influence muscle quality and functional recovery. This study aimed to examine longitudinal changes in muscle mass, muscle quality assessed by phase angle (PhA), and physical function after CPO and explored their postoperative interrelationships. **Methods**: This prospective longitudinal study included 35 female patients (mean age 34.9 ± 13.4 years) undergoing CPO. Assessments were conducted preoperatively, at full weight-bearing (FWB), and 12 months postoperatively. Lower-limb muscle mass and PhA were measured using multifrequency bioelectrical impedance analysis. Physical function was evaluated using Timed Up and Go (TUG), body weight-normalized ground reaction force during sit-to-stand (F/w), and operated-side weight-bearing capacity. **Results**: Operated-side muscle mass decreased at FWB and partially recovered by 12 months. In contrast, PhA declined markedly at FWB on the operated side (5.21 ± 0.69° to 4.15 ± 0.67°, *p* < 0.001) and remained significantly lower than baseline at 12 months. Functional measures declined during restricted loading and recovered to levels comparable to baseline by 12 months. At FWB, PhA was independently associated with TUG, F/w, and power generation indices. The reduction in PhA was greater than that observed for muscle mass, and contralateral PhA also declined. **Conclusions**: CPO is associated with a transient decline in muscle quality and functional performance during postoperative loading restriction. Although functional measures recover within 12 months, muscle quality remains partially impaired. Early strategies aimed at preserving muscle quality may support postoperative recovery.

## 1. Introduction

In a population-based cohort study in Johnston County, North Carolina, USA, including both men and women (approximately 54% women), a 25.3% lifetime risk of symptomatic osteoarthritis of the hip (OAhip) was observed among individuals aged 45–93 years [1]. In that cohort, the lifetime risk was higher in women (28.6%) than in men (18.5%). Similarly, epidemiological data from Japan indicate that OAhip is more prevalent among women [2]. Given this sex-related difference in epidemiology, the present study focused exclusively on Japanese female patients to ensure population homogeneity and reduce potential sex-related confounding.

**Figure 1:**
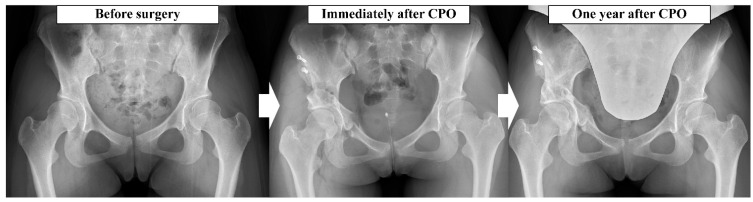
Changes in hip joint structure before and after CPO and 1 year after surgery. CPO changes the joint structure and reconstructs a stable hip joint. The changes in the hip joint structure before and after surgery and one year after surgery are shown, and it can be confirmed that the coverage of the hip joint has increased. CPO: curved periacetabular osteotomy.

Total hip arthroplasty (THA) effectively reduces pain and improves function [3]. However, given concerns regarding implant longevity in younger patients, periacetabular osteotomy (PAO) has been adopted as a joint-preserving procedure for acetabular dysplasia [4]. Several surgical techniques have been developed [5,6,7,8], and PAO has demonstrated long-term benefits, including improved postoperative activity levels [9] and hip survival, with approximately 60% of hips remaining free from conversion to THA at 20 years [10]. Curved PAO (CPO; Figure 1), developed by Naito et al. [7], reconstructs the acetabulum through a curved osteotomy, and secure bone union at the osteotomy sites has been demonstrated at 1 year postoperatively [11]. CPO is indicated for mild to severe dysplasia [12] and preserves blood supply to the rotated acetabular fragment, potentially reducing the risk of osteonecrosis [7]. Furthermore, preservation of the hip abductors has been associated with improvements in abductor strength and reduced dynamic instability during walking [13,14,15], which may facilitate early rehabilitation, with approximately 70% of patients participating in sports activities postoperatively [16].

Unlike patients undergoing THA, those undergoing CPO typically follow a period of non-weight-bearing or partial weight-bearing to protect the osteotomy, requiring staged rehabilitation. Several previous CPO studies have focused on bone morphology and healing [11], biomechanical alignment [17], and complications such as pubic nonunion [18]. However, the influence of postoperative weight-bearing restriction on soft tissue status, muscle function, and mobility performance remains insufficiently investigated. Reduced activity during restricted weight-bearing may contribute to declines in muscle mass, muscle quality, and fundamental movement performance; however, these relationships have not been fully characterized in the context of CPO.

Muscle function can be evaluated through muscle quantity and muscle quality. The phase angle (PhA), obtained via multifrequency bioelectrical impedance analysis (BIA), reflects cellular membrane integrity and intracellular fluid distribution. PhA has shown stronger associations with walking and standing performance than muscle mass in patients with severe OAhip awaiting THA [19].

Given its noninvasive nature and responsiveness, PhA may serve as a clinically meaningful indicator of functional recovery during postoperative rehabilitation.

Despite this potential utility, short- and mid-term changes in phase PhA, muscle mass, and physical function during postoperative weight-bearing restriction following CPO remain unclear. Additionally, the optimal rehabilitation targets and timing of intervention during early recovery phases have not been established.

Therefore, this study aimed to characterize longitudinal changes in lower-limb muscle mass, segmental lower-limb PhA, and performance-based functional measures after CPO, with a particular focus on the transition to full weight-bearing (FWB). The primary endpoint was the longitudinal change in lower-limb PhA as an indicator of muscle quality.

We hypothesized that (1) PhA would demonstrate a larger transient decline than muscle mass during the restricted-loading period and partially recover by 12 months, and (2) lower-limb PhA would be independently associated with functional performance after CPO.

## 2. Patients and Methods

### 2.1. Study Design and Measurement Parameters

This study was a prospective, longitudinal observational investigation with a 12-month follow-up after CPO. Assessments were performed at three standardized time points:

(1) Preoperatively, (2) at the FWB stage, and (3) 12 months postoperatively (12 months postoperatively).

FWB was authorized by the attending orthopedic surgeon based on postoperative timing (≥8 weeks), radiographic confirmation of bone union and stable acetabular position on plain radiographs, and patient-reported pain allowing for independent ambulation without an assistive device. All outcome assessments at the FWB stage were performed by licensed physical therapists who were not involved in the surgical decision-making process. The postoperative measurement timing was 115.5 ± 18.8 days for the FWB stage and 365.0 ± 21.2 days for 12 months postoperatively.

The following outcomes were evaluated: PhA, a bioelectrical impedance-derived index reflecting cellular membrane integrity and muscle quality; the Timed Up and Go (TUG) test, a widely used indicator of functional mobility; vertical ground reaction force normalized to body weight during the sit-to-stand task (F/w); normalized rate of force development (RFD8.75/w); and lower-limb loading during sit-to-stand movement.

### 2.2. Participants

Participants were patients who underwent CPO between December 2019 and June 2023 and completed all assessments at the three predefined time points during outpatient follow-up. Rehabilitation during hospitalization was standardized using the weight-bearing protocol described in Table 1.

Given that acetabular dysplasia is more prevalent among women [2], only female participants were included to reduce sex-related confounding. Additional inclusion criteria were established to ensure safe and reliable measurement conditions:

(1) Female sex;

(2) The absence of pain in anatomical regions other than the operated hip during weight-bearing, defined as a visual analogue scale (VAS; 0–100 mm) score of 0 mm in non-hip anatomical regions at each assessment;

(3) No history of osteotomy on the contralateral hip;

(4) The ability to ambulate independently;

(5) Independence in activities of daily living;

(6) No pacemaker implantation.

**Figure 2:**
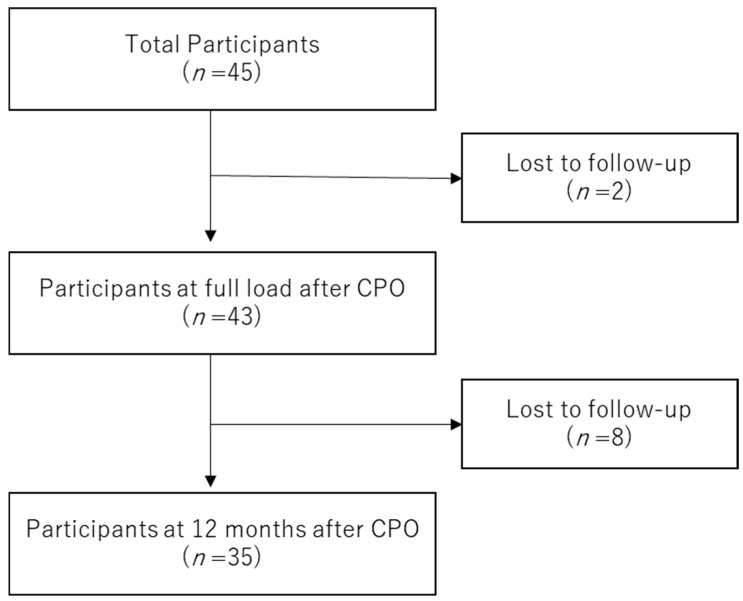
Of the 45 enrolled participants, 43 completed the FWB assessment (115.5 ± 18.8 days postoperatively), and 35 completed the 12-month assessment (365.0 ± 21.2 days). Two participants were lost before the FWB assessment and eight during follow-up. Abbreviations: CPO, curved periacetabular osteotomy; FWB, full weight-bearing.

Among the 45 patients who completed preoperative testing, 2 did not participate in outpatient rehabilitation at the FWB stage, and 8 discontinued follow-up before the 12-month assessment. Consequently, 35 patients with complete longitudinal data were included in the final analysis (Figure 2). Although preoperative testing was conducted in all 45 patients, complete longitudinal outcome data were not available for those who discontinued follow-up; therefore, a formal comparison between completers and dropouts was not performed.

### 2.3. CPO and Postoperative Rehabilitation Protocol

CPO was performed using the standardized technique described by Naito et al. [7]. The osteotomy was created around the anterior superior iliac spine, and the acetabular fragment was mobilized and repositioned to improve femoral head coverage.

The mobilized fragment was fixed using bioabsorbable screws. Fixation consisted of either four 6.5 mm screws or three 6.5 mm screws combined with one 4.5 mm screw, depending on intraoperative assessment of fragment stability. Although the fixation configuration was adjusted according to fragment stability, the surgical approach and osteotomy technique were consistent across all patients.

Postoperatively, patients followed a staged rehabilitation protocol. Partial weight-bearing was initiated according to clinical progress, and progression to FWB was permitted based on postoperative timing, radiographic confirmation of bone union, and improvement in pain during ambulation.

Bone union was primarily evaluated using plain radiographs at routine follow-up visits. When delayed union was suspected, computed tomography (CT) was additionally performed.

According to the postoperative weight-bearing protocol (Table 1), patients remained non-weight-bearing and used wheelchairs for the first 2 weeks. From postoperative weeks 2–4, 10 kg of weight-bearing was permitted using a wheelchair and crutches; from weeks 4–6, 20 kg of weight-bearing was allowed. During postoperative weeks 6–8, patients progressed to partial weight-bearing (one-half or two-thirds of body weight) using bilateral or single crutches. FWB was allowed after week 10, enabling a transition to independent gait. Although the protocol permitted FWB after postoperative week 8, the actual timing of FWB authorization was individualized based on clinical assessment and radiographic findings; therefore, the FWB stage assessment was performed at the outpatient visit when, after FWB was permitted, each patient was able to ambulate independently without an assistive device.

### 2.4. PhA and Muscle Mass Measurements

All measurements were performed in a controlled environment at the same facility using the same device and standardized body positioning. However, hydration status, fasting state, and time of day were not strictly standardized across participants. PhA and muscle mass were measured using a multifrequency, eight-electrode bioelectrical impedance analyzer (MC-780A-N, Tanita, Tokyo, Japan). Measurement procedures (including electrode placement, standing posture, and pre-measurement instructions) followed the standardized protocol described in our previous study [19].

Before measurement, electrode contact areas were cleansed with alcohol to ensure adequate conductivity. Participants stood barefoot on the foot electrodes, holding the hand electrodes with their arms positioned several centimeters away from the trunk. The analyzer delivered a low-level alternating current (<90 μA) to measure resistance (R) and reactance (Xc) across frequencies of 5, 50, and 250 kHz, allowing for an estimation of intracellular and extracellular water volumes.

PhA was calculated at 50 kHz using the following equation:

(1) $$PhA({}^{\circ})=arctan(\frac{Xc}{R})\times \frac{180}{\pi }$$

Muscle mass was quantified for each body segment and normalized to body weight to account for differences in body size. As in our previous study [19], inter-limb symmetry was evaluated by calculating the ratio of muscle mass and PhA of the operated limb to those of the contralateral limb.

### 2.5. TUG Test

The TUG test was administered according to the protocol developed by Podsiadlo and Richardson [20]. The test has demonstrated high inter-rater reliability [21].

Participants sat on a 42 cm high armless chair. At a verbal cue, they were instructed to stand up, walk 3 m, return, and sit down again. Two trials were performed, and the fastest time was recorded.

### 2.6. RFD8.75/w and F/w

Ground reaction force during sit-to-stand was measured using a motor performance evaluation device (ZaRitz, Tanita Corp., Tokyo, Japan) at a sampling frequency of 80 Hz and a resolution of 0.01 kgf/s/kg. Participants sat toward the front of a 42 cm high chair, positioned their feet 10 cm apart on embedded force sensors, crossed their arms over their chest, looked forward, and performed three maximal-effort sit-to-stand movements.

Ground reaction force during sit-to-stand has been associated with falls in community-dwelling older adults [22]. From the force–time curve, the following variables were extracted:

F/w: peak vertical ground reaction force normalized to body weight [19].

RFD8.75/w: the rate of force development calculated over an 87.5 ms window (corresponding to seven consecutive samples at a sampling frequency of 80 Hz) centered on the point of maximal slope, normalized to body weight. This analytical approach was adopted from our previously published methodology [19], which used the same sampling frequency and window length to quantify early-phase force development.

Operated-side weight-bearing (%): operated-side load divided by the sum of operated- and contralateral-side loads, expressed as a percentage.

For all indicators, the trial with the highest RFD8.75/w was used for analysis. Lower-limb loading ratios were calculated using the same trial.

### 2.7. Statistical Analyses

Statistical analyses were performed using SPSS version 29.0.1.0 (IBM Corporation, Armonk, NY, USA). Data normality was assessed using the Shapiro–Wilk test. Although minor deviations from normality were observed in some variables, Q–Q plots did not show substantial departures from normality; therefore, parametric analyses were applied consistently given the robustness of repeated-measures analysis of variance (ANOVA) and paired *t*-tests in moderate sample sizes (n = 35). Continuous variables are presented as the mean ± standard deviation. The unit of analysis was the individual patient (n = 35). Although limb-level measurements were obtained, comparisons between operated and contralateral limbs were performed within participants, and limbs were not treated as independent observations. Longitudinal analyses were also conducted at the patient level. Longitudinal changes across the three time points were examined using one-way repeated-measures analysis of variance (ANOVA). The assumption of sphericity was assessed using Mauchly’s test, and Greenhouse–Geisser corrections were applied when the assumption was violated. When a significant main effect of time was observed, post hoc pairwise comparisons were performed using paired *t*-tests with Bonferroni adjustment (α = 0.0167). Multiple regression analyses were conducted to identify factors associated with motor function variables (TUG, F/w, and RFD8.75/w). Independent variables included muscle mass, PhA, and lower-limb loading, selected based on previous research [19]. Stepwise selection was applied (entry *p* < 0.05, removal *p* > 0.10). Multicollinearity was assessed using the variance inflation factor. Model assumptions were evaluated as follows: linearity was assessed using scatterplots of standardized residuals versus predicted values; homoscedasticity was examined by visual inspection of residual dispersion; and normality of residuals was evaluated using Q–Q plots. Multicollinearity was assessed using variance inflation factors, with values < 5 considered acceptable. Model fit was assessed using R^2^ and adjusted R^2^. Statistical significance was set at *p* < 0.05. Because this was a prospective observational study conducted at a single center, an a priori sample size calculation was not performed.

## 3. Results

### 3.1. Participants and Postoperative Course

All participants were female (n = 35), with a mean age of 34.9 ± 13.4 years, height of 158.6 ± 5.23 cm, and body weight of 56.38 ± 8.31 kg at baseline. All participants were ambulatory and independent in activities of daily living.

### 3.2. Longitudinal Changes Across the Three Time Points and Contralateral Ratios (Table 2)

Body weight did not change significantly throughout the study period. Lower-limb muscle mass on the operated side decreased significantly at FWB and partially recovered at 12 months, whereas contralateral muscle mass did not show significant longitudinal change.

**Table 2:** Longitudinal changes after CPO.

Measurement Parameters	Pre (a)	FWB Stage (b)	After 12 Months (c)	Difference (*p* Value)		
				a and b	a and c	b and c
Body weight (kg)	56.38 ± 8.31	56.25 ± 8.13	56.60 ± 9.01	-	-	-
CPO-side lower-limb muscle mass (kg)	6.81 ± 0.70	6.43 ± 0.79	6.69 ± 0.82	<0.001 *	0.043	<0.001 *
Contralateral lower-limb muscle mass (kg)	6.85 ± 0.70	6.74 ± 0.70	6.77 ± 0.77	-	-	-
CPO-side lower-limb muscle mass/body weight (%)	12.21 ± 1.36	11.55 ± 1.43	11.98 ± 1.55	<0.001 *	0.024	<0.001 *
Contralateral lower-limb muscle mass/body weight (%)	12.29 ± 1.29	12.12 ± 1.36	12.13 ± 1.47	0.049	0.069	0.939
CPO-side lower-limb PhA (°)	5.21 ± 0.69	4.15 ± 0.67	4.72 ± 0.67	<0.001 *	<0.001 *	<0.001 *
Contralateral lower-limb PhA (°)	5.25 ± 0.62	4.87 ± 0.61	4.93 ± 0.61	<0.001 *	<0.001 *	0.259
Ratio of CPO-side leg muscle mass to contralateral-side leg (%)	99.35 ± 2.35	95.27 ± 4.13	98.75 ± 2.50	<0.001 *	0.083	<0.001 *
Ratio of CPO-side leg PhA to contralateral-side leg (%)	99.05 ± 4.10	85.00 ± 5.55	95.65 ± 4.29	<0.001 *	<0.001 *	<0.001 *
TUG (s)	5.72 ± 1.52	5.93 ± 1.13	5.24 ± 0.72	0.367	0.032	<0.001 *
F/w (kgf·kg^− 1^)	1.33 ± 0.09	1.27 ± 0.09	1.33 ± 0.10	<0.001 *	0.934	<0.001 *
RFD8.75/w (kgf/s·kg^− 1^)	11.1 ± 1.86	10.3 ± 1.60	11.68 ± 1.78	0.009 *	0.030	<0.001 *
CPO-side lower-limb load amount (%)	48.56 ± 4.07	43.55 ± 4.20	48.60 ± 3.55	<0.001 *	0.965	<0.001 *
Contralateral-side lower-limb load amount (%)	51.43 ± 4.07	56.44 ± 4.20	51.39 ± 3.55	<0.001 *	0.965	<0.001 *

Longitudinal changes are analyzed using one-way repeated-measures analysis of variance (ANOVA). When a significant main effect of time is detected, post hoc pairwise comparisons are performed using paired *t*-tests with Bonferroni correction (*p* < 0.0167). Values are presented as the mean ± standard deviation. Ratio variables represent percentages calculated as (CPO side value/contralateral side) × 100. Statistically significant difference after Bonferroni correction: * *p* < 0.0167. Abbreviations: CPO, curved periacetabular osteotomy; FWB, full weight-bearing; TUG, Timed Up and Go; F/w, ground reaction force normalized to body weight during sit-to-stand; RFD8.75/w, rate of force development over an 87.5 ms window normalized to body weight.

In contrast, PhA demonstrated a distinct temporal pattern. On the operated side, PhA significantly decreased from 5.21 ± 0.69° preoperatively to 4.15 ± 0.67° at the FWB stage (*p* < 0.001). Although PhA significantly improved to 4.72 ± 0.67° at 12 months postoperatively (*p* < 0.001 vs. FWB stage), it remained significantly lower than preoperative values (*p* < 0.001).

A similar trend was observed on the contralateral side: PhA significantly decreased from 5.25 ± 0.62° preoperatively to 4.87 ± 0.61° at FWB (*p* < 0.001) and was 4.93 ± 0.61° at 12 months. PhA at 12 months remained significantly lower than baseline (*p* < 0.001), whereas the difference between PhA at FWB and 12 months was not significant (*p* = 0.259).

Physical function measures showed a transient decline during the restricted weight-bearing phase. TUG showed no significant difference between baseline (5.72 ± 1.52 s) and FWB (5.93 ± 1.13 s; *p* = 0.367). At 12 months, TUG (5.24 ± 0.72 s) was significantly lower than at the FWB stage (*p* < 0.001), whereas the difference between baseline and 12 months did not reach the Bonferroni-adjusted significance level (*p* = 0.032). F/w decreased from 1.33 ± 0.09 preoperatively to 1.27 ± 0.09 at the FWB stage (*p* < 0.001) and returned to 1.33 ± 0.10 at 12 months (*p* < 0.001 vs. FWB stage; *p* = 0.934 vs. baseline). Operated-side weight-bearing (%) decreased from 48.56 ± 4.07% preoperatively to 43.55 ± 4.20% at the FWB stage (*p* < 0.001) and recovered to 48.60 ± 3.55% at 12 months (*p* < 0.001 vs. FWB stage; *p* = 0.965 vs. baseline).

RFD8.75/w decreased significantly from 11.1 ± 1.86 preoperatively to 10.3 ± 1.60 at the FWB stage (*p* = 0.009) and increased to 11.68 ± 1.78 at 12 months (*p* < 0.001 vs. FWB stage). No significant difference was observed between baseline and 12 months (*p* = 0.030).

Contralateral ratios showed greater asymmetry in PhA than in muscle mass. The muscle mass ratio decreased from 99.35 ± 2.35% preoperatively to 95.27 ± 4.13% at the FWB stage (*p* < 0.001) and partially recovered to 98.75 ± 2.50% at 12 months (*p* < 0.001 vs. FWB stage; *p* = 0.083 vs. baseline). In contrast, the PhA ratio decreased from 99.05 ± 4.10% preoperatively to 85.00 ± 5.55% at the FWB stage (*p* < 0.001) and improved to 95.65 ± 4.29% at 12 months (*p* < 0.001 vs. FWB stage; *p* < 0.001 vs. baseline).

Overall, longitudinal analysis indicated that the postoperative decline was more pronounced for PhA than for muscle mass. Although PhA improved after resuming FWB, it remained significantly lower than baseline at 12 months. In contrast, most functional measures returned to or approximated preoperative values by 12 months.

### 3.3. Differences Between the CPO Side and the Contralateral Side (Table 3)

Preoperatively, no significant differences were observed between limbs in muscle mass normalized to body weight (*p* = 0.131) or PhA (*p* = 0.206); however, operated-side loading was slightly lower than contralateral loading (48.56 ± 4.07% vs. 51.43 ± 4.07%, *p* = 0.045).

**Table 3:** Comparison of muscle mass, PhA, and contralateral ratios across postoperative time points.

		CPO Side	Contralateral Side	Difference (*p* Value)
Pre	lower-limb muscle mass/body weight (%)	12.21 ± 1.36	12.29 ± 1.29	0.131
	lower-limb PhA (°)	5.21 ± 0.69	5.25 ± 0.62	0.206
	lower-limb load amount (%)	48.56 ± 4.07	51.43 ± 4.07	0.045 *
Post FWB	lower-limb muscle mass/body weight (%)	11.55 ± 1.43	12.12 ± 1.36	<0.001 *
	lower-limb PhA (°)	4.15 ± 0.67	4.87 ± 0.61	<0.001 *
	lower-limb load amount (%)	43.55 ± 4.20	56.44 ± 4.20	<0.001 *
After 12 months	lower-limb muscle mass/body weight (%)	11.98 ± 1.55	12.13 ± 1.47	0.010 *
	lower-limb PhA (°)	4.72 ± 0.67	4.93 ± 0.61	<0.001 *
	lower-limb load amount (%)	48.60 ± 3.55	51.39 ± 3.55	0.026 *
		**Ratio of CPO-Side** **Leg Muscle Mass to** **Contralateral-Side Leg (%)**	**Ratio of CPO-Side** **Leg PhA to** **Contralateral-Side Leg (%)**	**Difference** **(*p* Value)**
Pre		99.35 ± 2.35	99.05 ± 4.10	0.568
FWB stage		95.27 ± 4.13	85.00 ± 5.55	<0.001 *
After 12 months		98.75 ± 2.50	95.65 ± 4.29	<0.001 *

Paired *t*-tests are used for comparisons between operated and contralateral limbs at each time point. A *p*-value < 0.05 is considered statistically significant. Values are presented as the mean ± standard deviation. Ratio variables represent percentages calculated as (CPO side/contralateral side) × 100. Statistically significant difference: * *p* < 0.05. Abbreviations: CPO, curved periacetabular osteotomy; PhA, phase angle; FWB, full weight-bearing.

At FWB, muscle mass normalized to body weight (*p* < 0.001), PhA (*p* < 0.001), and lower-limb loading (*p* < 0.001) were all significantly lower on the operated side than on the contralateral side.

At 12 months, muscle mass normalized to body weight (*p* = 0.010), PhA (*p* < 0.001), and lower-limb loading (*p* = 0.026) remained significantly lower on the operated side.

Specifically, operated-side muscle mass normalized to body weight changed from 12.21 ± 1.36% preoperatively to 11.55 ± 1.43% at FWB and 11.98 ± 1.55% at 12 months. PhA decreased from 5.21 ± 0.69° preoperatively to 4.15 ± 0.67° at FWB, partially recovering to 4.72 ± 0.67° at 12 months. Lower-limb loading decreased from 48.56 ± 4.07% preoperatively to 43.55 ± 4.20% at FWB and returned to 48.60 ± 3.55% at 12 months.

### 3.4. Multiple Regression Analyses (Table 4)

Multiple regression analyses were conducted to identify independent predictors of TUG, F/w, and RFD8.75/w at each time point (Table 4). Preoperatively, operated-side lower-limb load amount was independently associated with TUG and RFD8.75/w, whereas both muscle mass/body weight and PhA were independently associated with F/w. At the FWB stage, PhA was independently associated with TUG and RFD8.75/w and remained significantly associated with F/w together with muscle mass/body weight. At 12 months postoperatively, operated-side lower-limb load amount was independently associated with TUG and RFD8.75/w and remained significantly associated with F/w, while muscle mass/body weight retained an additional association with F/w.

**Table 4:** Multiple regression analysis for functional outcomes at each time point.

Time Point	Outcome	Predictor	B	SE	β	95% CI	*p* Value	R^2^	Adjusted R^2^
Baseline	TUG	lower-limb load amount (%)	−0.211	0.054	−0.566	−0.320 to −0.102	<0.001	0.321	0.300
	F/w	lower-limb muscle mass/body weight (%)	0.044	0.009	0.647	0.027 to 0.062	<0.001	0.517	0.487
		lower-limb PhA (°)	0.071	0.017	0.525	0.036 to 0.106	<0.001		
	RFD8.75/w	lower-limb load amount (%)	0.176	0.074	0.384	0.026 to 0.326	0.023	0.148	0.122
FWB stage	TUG	lower-limb PhA (°)	−0.709	0.266	−0.421	−1.250 to −0.167	0.012	0.177	0.152
	F/w	lower-limb PhA (°)	0.087	0.018	0.647	0.050 to 0.123	<0.001	0.442	0.407
		lower-limb muscle mass/body weight (%)	0.022	0.008	0.350	0.005 to 0.039	0.015		
	RFD8.75/w	lower-limb PhA (°)	1.209	0.358	0.507	0.482 to 1.937	0.002	0.257	0.235
After 12 Months	TUG	lower-limb load amount (%)	−0.070	0.033	−0.342	−0.138 to −0.002	0.044	0.117	0.090
	F/w	lower-limb load amount (%)	0.011	0.004	0.365	0.002 to 0.020	0.023	0.253	0.206
		lower-limb muscle mass/body weight (%)	0.021	0.01	0.321	0.001 to 0.042	0.044		
	RFD8.75/w	lower-limb load amount (%)	0.272	0.073	0.543	0.123 to 0.421	0.001	0.295	0.274

Abbreviations: TUG, Timed Up and Go; F/w, ground reaction force normalized to body weight during sit-to-stand; RFD8.75/w, rate of force development over an 87.5-ms window normalized to body weight; PhA, phase angle. Predictors are presented using full variable names. Only variables retained in the final multivariable regression models at each time point are shown.

## 4. Discussion

### 4.1. Main Findings

To date, longitudinal evidence examining changes in muscle mass, muscle quality assessed by PhA, and physical function following CPO appears to be limited. The present study provides prospective longitudinal data addressing this gap. Although operated-side muscle mass decreased significantly at FWB and partially recovered by 12 months, PhA demonstrated marked and persistent reductions, particularly during the FWB phase. On the operated side, PhA declined by approximately 20% from baseline (from 5.21 ± 0.69° to 4.15 ± 0.67°) and remained significantly lower than preoperative values even at 12 months.

In contrast, physical function—including TUG performance, F/w, and weight-bearing capacity—deteriorated temporarily during the restricted weight-bearing period, followed by recovery to preoperative levels by 12 months. Multiple regression analyses further indicated that PhA was independently associated with all functional outcomes at the FWB stage, highlighting the importance of muscle quality in early postoperative recovery. These findings suggest that although muscle mass decreased transiently after surgery and partially recovered by 12 months, muscle quality was more sensitive to the postoperative restricted loading environment and may influence early functional performance.

### 4.2. Changes from Preoperative to FWB

Before surgery, muscle mass and PhA were symmetrical between limbs, whereas operated-side weight-bearing was slightly lower than contralateral loading. At the FWB stage, operated-side muscle mass decreased significantly, and PhA exhibited a marked decline. These changes were accompanied by significant reductions in F/w and weight-bearing capacity, whereas TUG did not show a significant difference compared with baseline.

The decline in PhA and functional indices is likely multifactorial. Reduced activity during the non-weight-bearing and partial-weight-bearing phases after CPO may limit muscle activation, particularly on the operated side. Patients often rely on wheelchairs early after surgery, and subsequent gait affected by the crutches restricts lower-limb loading. A previous study reported that the use of canes reduces hip muscle activity [23], supporting the possibility that postoperative mobility patterns contribute to a decline in muscle quality.

The greater magnitude and persistence of reduction in PhA compared with muscle mass aligns with prior work showing lower PhA in the affected limbs of patients with OAhip [19] and in pre-frail older adults [24]. The present findings suggest that reduced loading after CPO may influence not only the operated limb but also, to a lesser extent, the contralateral side. PhA may therefore serve as a sensitive indicator of early changes in muscle quality associated with restricted loading and decreased habitual activity. Monitoring PhA during this period may help clinicians identify patients at risk of prolonged muscle quality deficits and guide early preventive interventions.

Notably, contralateral PhA declined despite the absence of significant changes in contralateral muscle mass. This dissociation between muscle quantity and quality suggests that postoperative adaptations were not solely attributable to localized surgical trauma. Rather, reduced overall physical activity, bilateral unloading during the non-weight-bearing phase, and possible systemic inflammatory responses may have contributed to generalized muscle quality deterioration. Although the magnitude of decline on the contralateral side was lower than that observed on the operated limb, its presence indicates that early postoperative changes likely reflect a combination of localized mechanical effects and broader systemic or behavioral influences.

### 4.3. Changes from Full Weight-Bearing to 12 Months Postoperation

Between FWB and 12 months, most functional measures showed significant improvement and returned to levels comparable to baseline, whereas PhA on the operated side demonstrated only partial recovery. Restoration of joint structure achieved by CPO likely contributed to these functional improvements. Increased acetabular coverage after CPO may enhance hip joint stability [15], facilitating more efficient force generation during gait and functional tasks. Preservation of the hip abductor musculature—an inherent advantage of CPO—has been associated with improved abductor strength and reduced dynamic instability during walking [13,14,15]. These mechanisms may help explain the improvement in TUG observed between FWB and 12 months.

However, despite recovery in muscle mass and function, PhA remained below preoperative levels. This suggests that although mechanical stability and functional capacity improved, the prolonged period of reduced loading may continue to influence muscle quality. Regression analyses at 12 months indicated that weight-bearing capacity was associated with all functional variables, suggesting that promoting safe, progressive loading after bone union may facilitate further recovery in muscle activity and PhA. Given that PhA improved from approximately a 20% decline at FWB to roughly a 10% decline at 12 months, longer follow-up may reveal whether muscle quality continues to normalize over time.

### 4.4. Clinical Implications

At 12 months postoperatively, physical function had recovered to or exceeded preoperative levels, supporting the effectiveness of CPO in restoring functional mobility alongside structural realignment. However, several functional indicators, including F/w, weight-bearing capacity, and RFD, declined significantly during the restricted weight-bearing phase, indicating that early postoperative management represents a critical period in recovery. In particular, the non-weight-bearing and partial-weight-bearing phases, as well as the transition to FWB, appear to represent critical windows for intervention. These periods correspond to the greatest decline in PhA and temporary deterioration in several functional measures. Moreover, because PhA was independently associated with functional outcomes at the FWB stage, interventions targeting muscle quality during and prior to this transition may be especially relevant for optimizing early recovery.

PhA was independently associated with all functional outcomes at the FWB phase. Notably, PhA declined substantially during this period (operated side: 5.21 ± 0.69° to 4.15 ± 0.67°; contralateral side: 5.25 ± 0.62° to 4.87 ± 0.61°; Table 2) and remained lower than baseline even at 12 months. These findings suggest that muscle quality may play an important role in early postoperative mobility.

Although the present study did not directly evaluate exercise or nutritional interventions, prior research has reported that aerobic exercise [25], resistance training [26], and protein supplementation combined with resistance exercise [27] can improve indices of muscle quality. Therefore, these strategies may be considered part of postoperative rehabilitation planning, particularly during phases of restricted loading, and warrant investigation in future interventional studies.

Regarding the clinical significance, the improvement in TUG from baseline to 12 months was approximately 0.48 s. Although statistically significant, this magnitude of change is smaller than previously reported minimal clinically important difference (MCID) thresholds for TUG (approximately 1.4 s in patients with OAhip) [28] and smaller than minimal detectable change (MDC) values (approximately 1.1 s in individuals with knee osteoarthritis) [29]. Therefore, the observed TUG change should be interpreted cautiously in terms of clinical impact. In contrast, the decline in operated-side PhA at the FWB stage represented an approximate 20% reduction from baseline (5.21 to 4.15°), indicating a marked physiological alteration in muscle quality during the restricted loading phase. Although an established MCID for PhA is not currently available, the magnitude and consistency of this decline, together with its independent association with functional performance at the FWB stage, suggest that it may represent a clinically meaningful signal warranting further investigation.

As patients progress toward partial loading, transitioning from open kinetic chain to closed kinetic chain exercises (e.g., half-weight-bearing squats) may be reasonable from a rehabilitation perspective, although this was not directly tested in the present study. Physical activity has been shown to correlate positively with PhA [30]; thus, gradual and clinically appropriate increases in activity may be beneficial. Given that approximately 70% of patients participate in sports activities following CPO [16], strategies aimed at minimizing early PhA decline may warrant further investigation on long-term athletic and high-level functional outcomes.

### 4.5. Limitations and Future Directions

This study has some limitations. First, only female patients were included, which limited the generalizability of the findings, particularly to male patients with hip dysplasia. In addition, although all participants underwent preoperative testing, complete longitudinal outcome data were not available for those who discontinued follow-up; therefore, a formal comparison between completers and dropouts was not performed. As a result, potential selection bias cannot be excluded, and the findings may preferentially reflect outcomes in patients who adhered to the full follow-up protocol.

Second, the observational design precludes causal inference. Although significant associations were observed between PhA and functional outcomes at the FWB stage, interventional studies are needed to determine whether improving muscle quality leads to improvements in physical function after CPO.

Third, the timing of FWB varied depending on clinical judgment, which may have influenced recovery trajectories. More standardized timing in future studies may reduce variability.

Fourth, PhA was measured using BIA, which can be influenced by hydration, food intake, and daily fluctuations. Employing stricter measurement protocols or validating findings with imaging modalities such as magnetic resonance imaging or CT would strengthen conclusions. In addition, PhA can be influenced by short-term changes in hydration status and postoperative edema or inflammation, which were not directly quantified in this study.

Fifth, evaluators were not blinded to measurement timing, introducing potential detection bias. Blinded assessments should be considered in future studies.

Sixth, adherence to supervised rehabilitation and home exercise programs was not quantified, although such factors could influence functional recovery. Future studies should track adherence to better account for this variable. Pain severity, analgesic use, and objectively measured physical activity were not collected, and these factors may have influenced both PhA and functional performance.

Seventh, given the limited sample size, although three theoretically relevant predictors (lower-limb load amount, PhA, and lower-limb muscle mass/body weight) were considered in the regression analyses and the final models retained a maximum of two variables, formal model validation procedures such as cross-validation or bootstrapping were not performed. Therefore, these findings should be interpreted with caution given the modest sample size and single-center design, and should be validated in larger, preferably multicenter cohorts to enhance generalizability.

Finally, although functional outcomes recovered by 12 months, PhA did not return to baseline. A longer follow-up is needed to determine whether muscle quality continues to improve and to identify factors that facilitate sustained recovery.

As a reference for planning future studies, assuming a two-tailed α = 0.05 and 80% power for a within-subject (paired) comparison, approximately 34 participants would be required to detect a moderate standardized effect size (Cohen’s dz = 0.50). Larger samples (approximately 52 and 90 participants) would be required to detect smaller effect sizes (dz = 0.40 and 0.30, respectively).

## 5. Conclusions

This longitudinal study demonstrated that although physical function temporarily declined during the postoperative weight-bearing restriction period after CPO, it recovered to levels comparable to baseline by 12 months postoperatively. In contrast, operated-side muscle mass decreased transiently during the restricted loading phase and partially recovered by 1 year, whereas PhA, an indicator of muscle quality, significantly declined after surgery and remained below baseline at 12 months. Importantly, PhA was independently associated with key functional outcomes, such as mobility and power generation, particularly during the FWB stage. These findings suggest that preserving muscle quality during the early postoperative phase may play an important role in functional recovery after CPO. Rehabilitation strategies may therefore consider incorporating early, safe loading and individualized interventions to mitigate PhA decline. Further longitudinal studies with larger cohorts and extended follow-up are warranted to confirm these observations.

## Figures and Tables

**Table 1:** Weight-bearing and lifestyle after CPO.

Postoperative Period (Weeks)	Weight on CPO Side	Lifestyle	Rehabilitation Program
0–2	No load	Wheelchair	Basic movement exercises, range of motion exercises, low-impact muscle strengthening exercises
2–4	10 kg load	Wheelchair and both crutches	10 kg weight training, crutch training, range of motion training, low-load muscle strengthening training
4–6	20 kg load	Wheelchair and both crutches	20 kg weight training, crutch training, range of motion training, low-load muscle strengthening training
6–8	1/2 or 2/3 of body weight	Both crutches or one crutch	2/3 body weight loading training, crutch training, range of motion training, muscle strengthening training
>10	full weight bearing	One crutch or no walking aid	2/3 body weight or full loading training, range of motion training, muscle strengthening training

Abbreviations: CPO, curved periacetabular osteotomy.

## Data Availability

The data presented in this study are only available upon request from the corresponding author due to ethical restrictions.

## Abbreviations

The following abbreviations are used in this manuscript:

CPO	curved periacetabular osteotomy
PAO	periacetabular osteotomy
THA	total hip arthroplasty
FWB	full weight-bearing
PhA	phase angle
BIA	bioelectrical impedance analysis
TUG	Timed Up and Go
F/w	ground reaction force normalized to body weight during sit-to-stand
RFD8.75/w	rate of force development over an 87.5 ms window normalized to body weight
